# Abnormal Orientation of the Superior Mesenteric Vessels Detected in Asymptomatic Infants: What Is Its Destiny?

**DOI:** 10.3389/fped.2021.665448

**Published:** 2021-06-11

**Authors:** Joonhyuk Son, Sanghoon Lee, Wontae Kim, Soo-Min Jung, Tae Yeon Jeon, So-Young Yoo, Ji Hye Kim, Jeong-Meen Seo

**Affiliations:** ^1^Department of Pediatric Surgery, Hanyang University College of Medicine, Seoul, South Korea; ^2^Department of Surgery, Samsung Medical Center, Sungkyunkwan University School of Medicine, Seoul, South Korea; ^3^Department of Radiology, Samsung Medical Center, Sungkyunkwan University School of Medicine, Seoul, South Korea

**Keywords:** malrotation, midgut volvulus, ultrasonography, mesenteric vessels, abnormal orientation

## Abstract

**Background:** Ultrasonography (USG) has been described as an alternative diagnostic tool for malrotation that evaluates the orientation of the superior mesenteric vessels. However, literature concerning the management of patients who do not have abdominal symptoms is limited. We aimed to review the clinical course of infants showing abnormal orientation of the superior mesenteric vessels on USG who were asymptomatic at the time of diagnosis.

**Methods:** Seventy asymptomatic infants with abnormal orientation of the superior mesenteric vessels in a single center between 2014 and 2018 were retrospectively analyzed.

**Results:** The 70 patients, 21 underwent upper gastrointestinal series (UGIS) and 11 underwent abdominal surgery for other surgical conditions. Among the 32 (45.7%) patients who underwent UGIS or abdominal surgery, 11 were proven to have malrotation. Of the 38 (54.3%) patients who did not undergo UGIS or abdominal surgery, six patients were too unstable to undergo UGIS, five died due to cardiac complications, and the remaining patient developed midgut volvulus and died 3 days after emergency surgery. The remaining 32 patients who did not undergo UGIS or abdominal surgery were discharged without additional tests, and all were asymptomatic until their last follow-up. In the multivariate analysis, history of heart surgery and the presence of more than three anomalies were significantly associated with malrotation.

**Conclusion:** A significant number of malrotation were diagnosed in asymptomatic infants with abnormal orientation of the superior mesenteric vessels on USG. Infants with major cardiac or multiple anomalies need special attention and should undergo UGIS in a promptly manner to confirm malrotation.

## Introduction

Malrotation in newborns can lead to conditions such as midgut volvulus, which may result in grave complications when timely surgical interventions are not undertaken. Many studies suggest upper gastrointestinal series (UGIS) as the diagnostic test of choice for malrotation, while others have used ultrasonography (USG) to evaluate the orientation of the superior mesenteric artery (SMA) and vein (SMV) (1–4). Abnormal orientation of the superior mesenteric vessels is a rare but notable finding on abdominal USG in infants. However, abnormal orientation of the superior mesenteric vessels does not always correlate with malrotation (5–7). It is obvious that prompt evaluation including imaging studies should be performed in infants with suspicious symptoms suggesting malrotation with volvulus, such as bilious vomiting, abdominal distension, and feeding intolerance. However, for asymptomatic infants with an incidental finding of abnormal SMV and SMA orientation on abdominal USG, there are no established protocols regarding their diagnostic and treatment strategies. For these reasons, we reviewed our center's data on infants with abnormal orientation of the superior mesenteric vessels on USG who were asymptomatic at the time of USG.

## Methods

**Figure 1:**
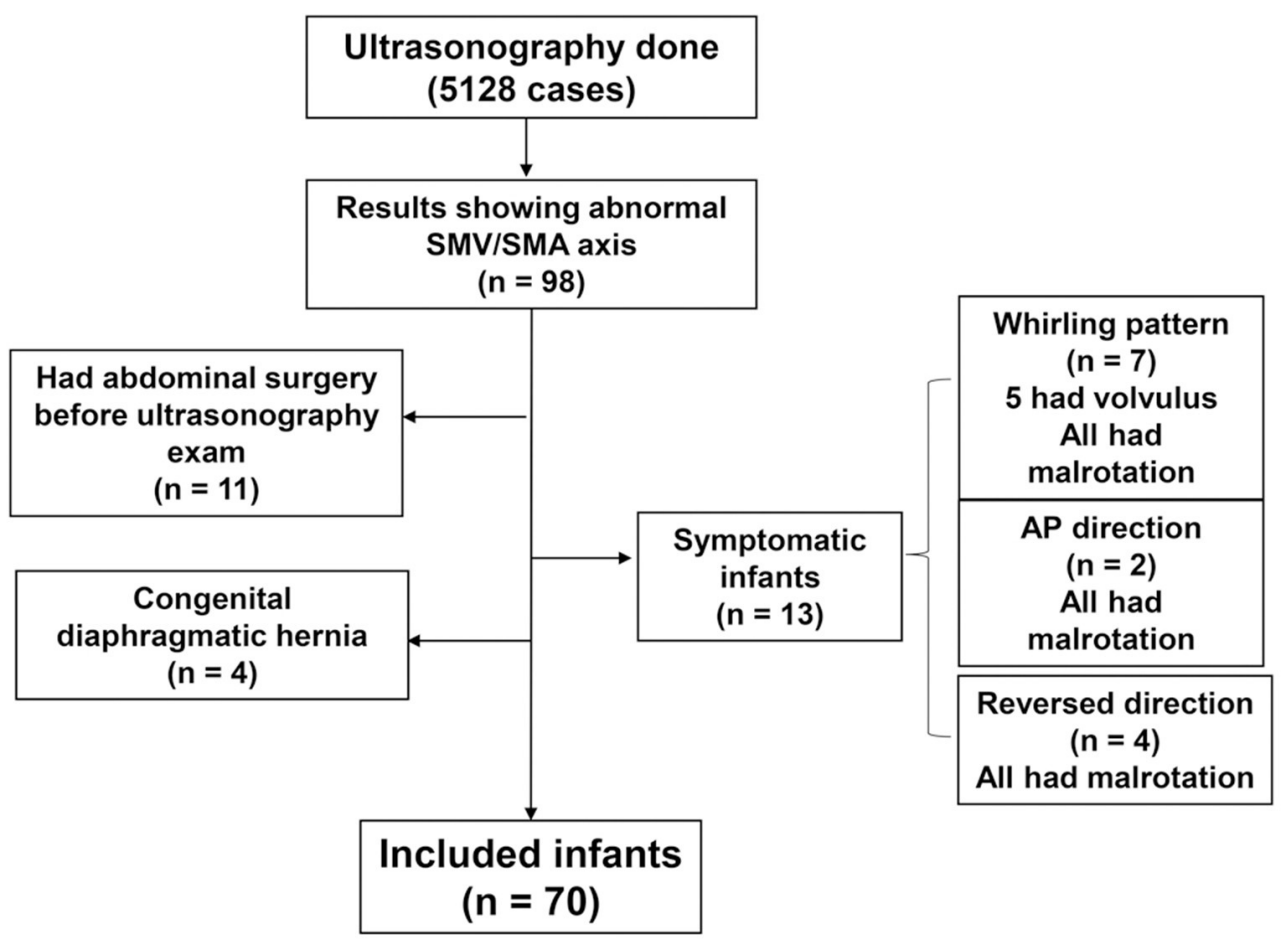
Flowchart of the study (inclusion criteria).

All ultrasonographic findings of infants <12 months old at Samsung Medical Center (Seoul, South Korea) between January 2014 and December 2018 were retrospectively reviewed. All USG results with a description of the abnormal orientation of the superior mesenteric vessels were included in the study. Infants with abdominal symptoms such as vomiting, abdominal distension, and feeding intolerance at the time of USG were excluded. It is well-known that patients with gastroschisis, omphalocele, or congenital diaphragmatic hernia are associated with malrotation (8); thus, they were excluded from the study. Patients who underwent abdominal surgery before USG were also excluded because manipulation of the intestines and their mesenteries during surgery may present with seemingly abnormal orientation of the mesenteric vessels. A flowchart regarding patient inclusion is described in Figure 1.

**Figure 2:**
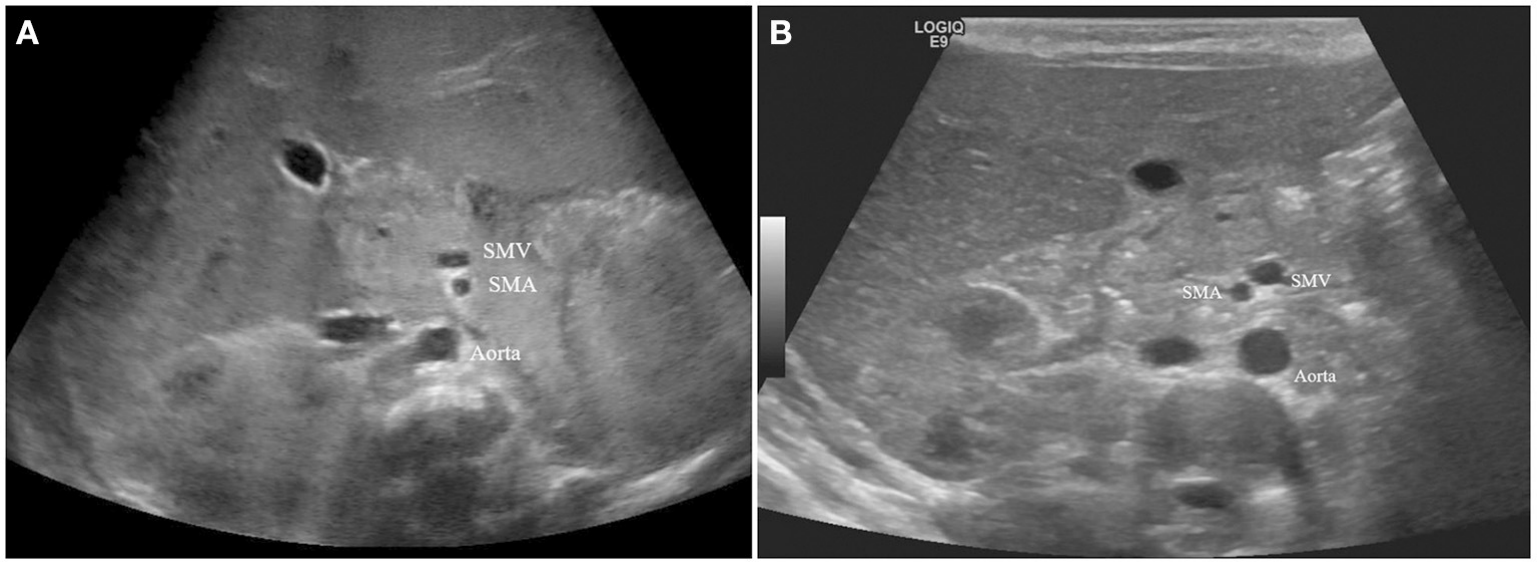
**(A)** Anterior/Posterior direction of SMV and SMA. **(B)** Reversed direction of SMV and SMA.

Examination and description of the orientation of the superior mesenteric vessels was a routine component of the USG procedure in infants at our center during the study period. All USG procedures were performed by experienced radiologists who specialize in pediatric radiology. The orientation of the SMV and SMA was determined at the level of the proximal SMV and SMA by reviewing USG images and classified into the following categories: when the SMV was on the anterior side of the SMA, it was classified as anterior/posterior (AP) direction (Figure 2A); when the SMV was on the left side of the SMA, it was classified as a reversed direction (Figure 2B); normal position was when the SMV was on the right side of the SMA. Intestinal rotation was considered normal when the position of duodenojejunal junction was left of the left vertebral body pedicle at the level of the duodenal bulb at UGIS. All other cases were considered malrotations.

Patients who were discharged were scheduled for the outpatient clinic every 1–2 months until they are > 12 months old. Detailed medical history was obtained, and physical examination was performed at every clinic visits.

We reviewed the medical records of patients who were included in the study and analyzed their clinical courses. A chi-squared test was used to compare categorical variables, and a Student's *t*-test was used for continuous variables. Logistic regression analysis was performed for multivariate analysis. *P* < 0.05 were considered statistically significant.

This study was approved by the Institutional Review Board at Samsung Medical Center (IRB File No. 2020-11-100).

## Results

### Patient Characteristics

A total of 5,128 USG results were reviewed, and 70 cases in which the USG report showed abnormal orientation of the superior mesenteric vessels (AP or reversed direction) were included in the study. There were 59 patients in the AP direction and 11 in the reversed direction. Demographic characteristics of the patients are shown in Table 1. The median age at USG was 7 (range, 0–182) days and the mean birthweight of the infants was 2,340 ± 909 g. The median follow-up duration was 506 (7–1,810) days, and the median age at the time of last follow-up was 513 (13–1,915) days. Most characteristics were not statistically different between the AP and reversed direction groups; however, more patients in the reversed direction group had undergone UGIS or surgery than in the AP direction group (81.8 vs. 40.7%; *p* = 0.012).

**Table 1:** Patient demographics.

	**Overall (*n =* 70)**	**AP direction (*n* = 59)**	**Reversed (*n =* 11)**	***p***
Gestational age (weeks, mean)	35^+4^wks	35^+5^wks	34^+6^wks	0.551
Birth weight (g, mean ± SD)	2,340 ± 909	2,385 ± 898	2,095 ± 977	0.335
Sex				
Male (%)	28 (40.0)	25 (42.4)	3 (27.3)	0.506
Female (%)	42 (60.0)	34 (57.6)	8 (72.7)	
Age at ultrasonography (days, median)	7 (0–182)	7 (0–153)	21 (0–182)	0.539
GI anomaly (%)	15 (21.4)	11 (18.6)	4 (36.4)	0.233
Cardiac anomaly requiring open heart Surgery (%)	23 (32.9)	18 (30.5)	5 (45.5)	0.485
VACTERL (%)	4 (5.7)	4 (6.8)	0 (0.0)	0.999
Multiple anomalies (%)	10 (14.3)	8 (13.6)	2 (18.2)	0.652
UGIS done (%)	21 (30.0)	17 (28.8)	4 (36.4)	0.723
Abdominal surgery done (%)	19 (27.1)	13 (22.0)	6 (54.5)	0.058
UGIS or surgery done (%)	33 (47.1)	24 (40.7)	9 (81.8)	0.012
Malrotation diagnosed (%)	12 (17.1)	9 (15.3)	3 (27.3)	0.386
Follow-up duration (days, median)	506 (7–1,810)	465 (7–1,810)	619 (63–1,224)	0.997
Age at last follow up (days, median)	513 (13–1,915)	469 (13–1,915)	693 (65–1,245)	0.956

*SD, standard deviation; GI, gastrointestinal; VACTERL, vertebral anorectal cardiac tracheoesophageal fistula renal radial limb; UGIS, upper gastrointestinal series*.

### Management Flow of the Patients With Abnormal Orientation of the Superior Mesenteric Vessels

**Figure 3:**
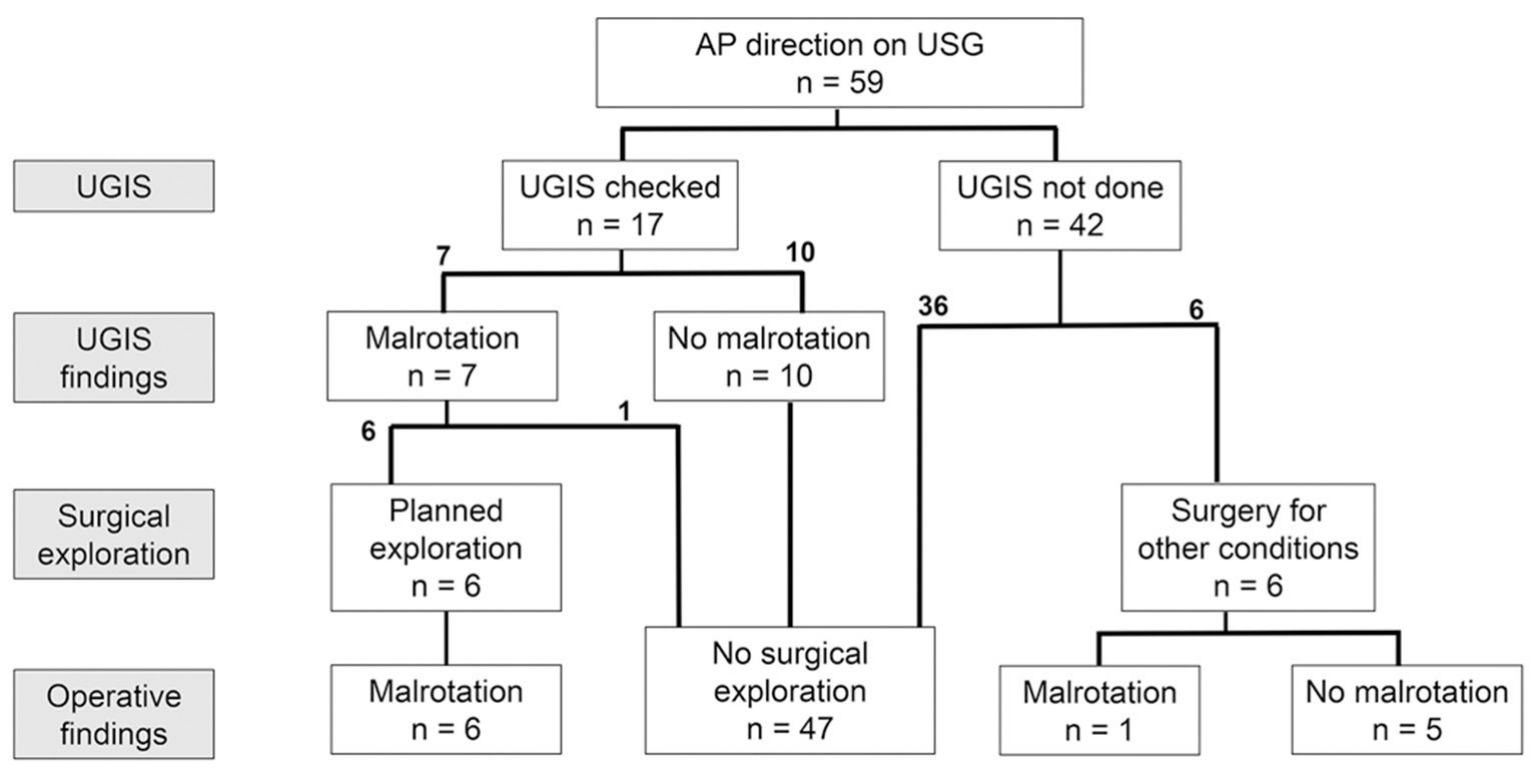
Management flow of the AP direction group.

The management flow of patients in the AP direction group is described in Figure 3. Seventeen patients in the AP direction group underwent UGIS. Of the 17 patients, seven patients showed malrotation, six of which underwent planned exploratory surgery for malrotation. Parents of one patient refused surgery, and close observation with outpatient visits was done for this patient. Six patients underwent abdominal surgery for other surgical conditions, and 36 patients did not undergo either UGIS or abdominal surgery. Among the six patients who underwent abdominal surgery for other surgical conditions, one patient was proven to have malrotation and underwent an additional Ladd procedure at the time of surgery.

**Figure 4:**
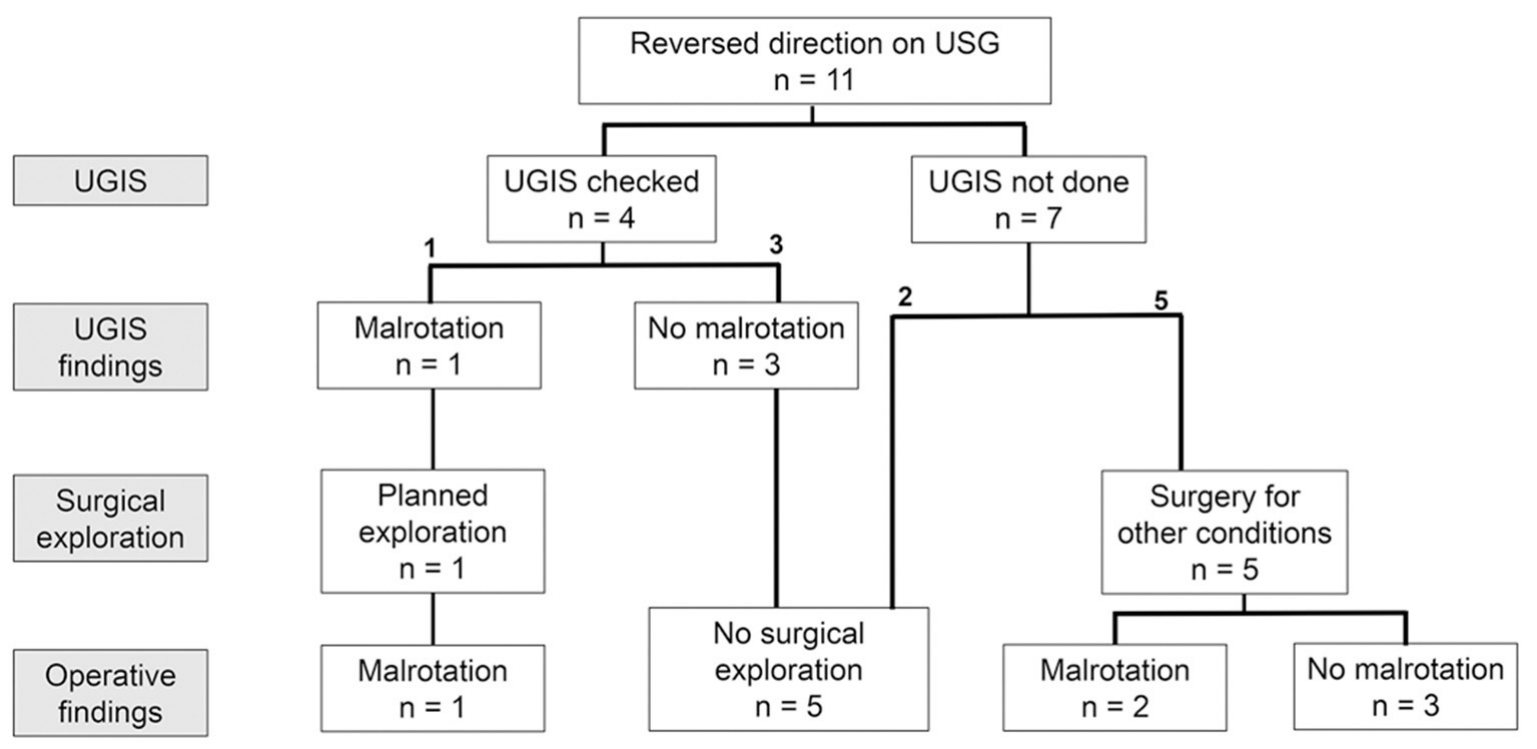
Management flow of the reversed direction group.

The management flow of patients in the reversed direction group is described in Figure 4. Of the 11 patients with reversed orientation of the superior mesenteric vessels, four underwent a UGIS study and one patient was diagnosed with malrotation. Five patients underwent an abdominal surgery for other reasons and two patients were proven to have malrotation.

### Clinical Outcomes of All Patients

**Figure 5:**
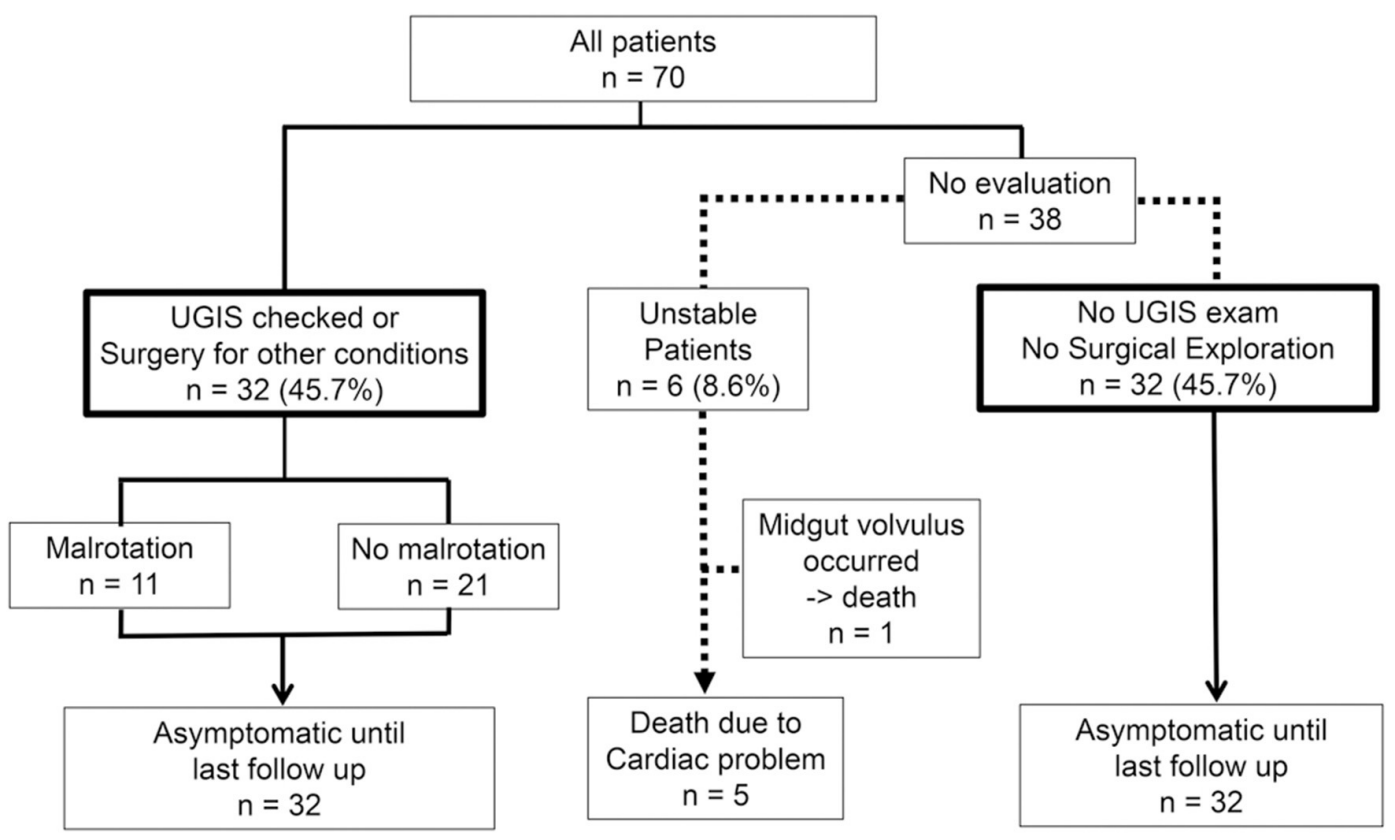
Clinical outcomes of all patients.

In total, 32 (45.7%) patients underwent UGIS or abdominal surgery (Figure 5). Among them, 11 patients were proven to have malrotation. All 32 patients had no symptoms until their last follow-up.

The other 38 patients (54.3%) did not undergo UGIS or surgical exploration. Of those, five died during hospitalization due to cardiac complications. One patient developed midgut volvulus 39 days after USG and underwent emergency surgery. The patient died 3 days after surgery due to multiorgan failure. The remaining 32 (45.7%) patients had no symptoms during their hospitalization and were discharged without confirmatory UGIS examinations. All 32 patients had no symptoms until their median age at the time of last follow-up was 763 (range, 46–1,915) days.

### Clinical Factors Associated With Malrotation

Logistic regression analysis of clinical factors for the presence of malrotation among the infants who underwent UGIS or abdominal surgery is shown in Table 2. History of heart surgery, and the presence of multiple anomalies (>3 major anomalies in one infant) were significant factors associated with malrotation in the univariate analysis. In the multivariate analysis, both variables remained as significant factors associated with malrotation (*p* = 0.016 and *p* = 0.021, respectively).

**Table 2:** Logistic regression analysis of clinical factors for malrotation.

**Variables**	**Univariate analysis**		**Multivariate analysis**	
	**OR (95% CI)**	***P***	**Adjusted OR (95% CI)**	***p***
Gestational age	1.096 (0.837–1.435)	0.507		
Birthweight	1.000 (0.999–1.001)	0.991		
Male (vs. female)	1.500 (0.342–6.583)	0.591		
AP direction (vs. reversed)	0.833 (0.166–4.184)	0.825		
GI anomaly	1.429 (0.331–6.170)	0.633		
VACTERL	6.667 (0.607–73.195)	0.121		
Cardiac anomaly requiring open heart surgery	6.400 (1.338–30.606)	0.020	16.562 (1.687–162.592)	0.016
Multiple anomalies	6.000 (1.134–31.735)	0.035	17.262 (1.539–193.570)	0.021

*OR, odds ratio; CI, confidence interval; AP, anterior/posterior direction; GI, gastrointestinal; VACTERL, vertebral anorectal cardiac tracheoesophageal fistula renal radial limb*.

## Discussion

The presentation time varies among patients with intestinal malrotation. According to previous literature, the peak age of presentation is the 1st month of life, and the majority of patients present within the 1st year, while some may never have any symptoms (8–10). For patients with symptoms or signs suggesting malrotation, the protocol for prompt evaluation and management is well-established in most institutions. However, the management flow of asymptomatic patients with malrotation is still under debate. There have been several studies on asymptomatic malrotation, but none have published definitive management protocols (11–15).

For decades, several authors have proposed abnormal orientation of the superior mesenteric vessels on USG as a finding of intestinal malrotation. As a result, incidental findings of abnormal orientation of the SMV and SMA are occasionally reported. However, the accuracy of abnormal orientation of the superior mesenteric vessels for diagnosing malrotation is low, especially when the patient is asymptomatic (3). Thus, abnormal USG findings could be seen in patients with normal intestinal rotation. Conversely, patients with malrotation could also show normal orientation of superior mesenteric vessels (5, 6, 16–18); therefore, UGIS is usually recommended for these patients to confirm malrotation. However, there is minimal evidence indicating a need for additional UGIS in all asymptomatic patients with abnormal SMV/SMA orientation found incidentally on USG. There have been several retrospective studies and systematic reviews regarding the clinical outcomes of infants with asymptomatic malrotation; however, most studies were focused on patients with cardiac anomalies, especially in heterotaxy syndromes (19–22). To the best of our knowledge, this is the first study to review the clinical outcomes of all asymptomatic infants with or without congenital anomalies who have incidental findings of abnormal SMV/SMA orientation on USG.

Whether to proceed with further evaluation for asymptomatic infants with abnormal USG findings is a controversial issue. There are currently no protocols or recommendations regarding the evaluation of asymptomatic patients with abnormal SMV/SMA orientation on USG. Due to the low accuracy of USG for diagnosing malrotation, it is rational to perform UGIS in these infants to confirm malrotation. It has been the policy in our center to recommend UGIS to all infants with incidental findings of abnormal SMV/SMA orientation. However, not all infants undergo UGIS due to various circumstances, such as poor patient condition and parental refusal. When UGIS is not performed in asymptomatic infants with abnormal SMV/SMA orientation, the parents are carefully counseled regarding the risk of malrotation and volvulus, with emphasis on the symptoms and signs of volvulus. The infants are then scheduled for regular clinic visits until they are >12 months old.

According to our results, 32 (45.7%) infants were discharged without confirmatory UGIS study, all of whom were asymptomatic until their last follow-up. From this one might infer that universal screening with UGIS is not warranted in asymptomatic infants with abnormal orientation of the superior mesenteric vessels on USG. However, our study has shown a positive predictive value of 36.4% for diagnosing malrotation with USG in asymptomatic infants, including patient who developed midgut volvulus. Orzech et al. (3) reported 32 (42.1%) out of 76 children (including symptomatic children) with abnormal USG findings had malrotation. Although our study included only asymptomatic infants, the positive predictive rate for diagnosing malrotation was comparable to the study by Orzech et al. Thus, all infants with abnormal orientation of superior mesenteric vessels, with or without abdominal symptoms, are at risk of having malrotation. Our multivariate analysis showed that a history of heart surgery and multiple anomalies were significant risk factors for malrotation in asymptomatic patients with abnormal USG findings. These factors are worth considering when the clinician must decide whether to perform further diagnostic or therapeutic interventions. Based on our results, infants with multiple anomalies or a major cardiac anomaly should undergo UGIS as soon as the patients' condition is stable enough to undergo examination.

Previous studies reported that the reversed direction of the superior mesenteric vessels was more predictive for diagnosing malrotation than the AP direction (1, 3, 6). However, this was inconsistent in our study. AP and reversed direction showed no statistical difference (37.5 vs. 33.3%, *p* = 0.825) in predicting malrotation. We assume that this difference occurred because we included only asymptomatic patients, whereas other studies performed analysis including symptomatic patients. The incidence of malrotation in patients with reversed direction in our center (33.3%) was lower than the other studies (100%) which included only symptomatic patients (1, 2, 6). We may infer that the reversed orientation of the superior mesenteric vessels is more predictive for diagnosing malrotation when the abdominal symptoms are accompanied.

The positive predictive value for diagnosing malrotation with USG may vary among institutions due to the different technical skills of radiologists and standards for evaluating the orientation of mesenteric vessels with USG. Thus, each institution should review their center's data of asymptomatic patients with abnormal orientation of the superior mesenteric vessels and establish their own management algorithm to select the best decision for these patients.

Our study has several limitations. First, it was a retrospective study with a relatively small number of cases, and few infants were followed up shortly. Second, we had no specific algorithm for determining whether patients should undergo UGIS or not, and several patients did not undergo UGIS or surgery to confirm malrotation; thus, our results may be biased. Additional prospective data should be collected to reach a consensus for the management plan of these patients in the future.

## Conclusion

A significant number of malrotation is seen in asymptomatic infants with abnormal orientation of the superior mesenteric vessels on USG. Infants with major cardiac or multiple anomalies have greater risk of malrotation and volvulus, thus further evaluation with UGIS is warranted.

## Data Availability Statement

The original contributions presented in the study are included in the article/supplementary material, further inquiries can be directed to the corresponding author/s.

## Ethics Statement

The studies involving human participants were reviewed and approved by Institutional Review Board at Samsung Medical Center (IRB File No. 2020-11-100). Written informed consent from the participants' legal guardian/next of kin was not required to participate in this study in accordance with the national legislation and the institutional requirements.

## Author Contributions

All authors listed have made a substantial, direct and intellectual contribution to the work, and approved it for publication.

## Conflict of Interest

The authors declare that the research was conducted in the absence of any commercial or financial relationships that could be construed as a potential conflict of interest.
